# Latest Evidence Regarding the Effects of Photosensitive Drugs on the Skin: Pathogenetic Mechanisms and Clinical Manifestations

**DOI:** 10.3390/pharmaceutics12111104

**Published:** 2020-11-17

**Authors:** Flavia Lozzi, Cosimo Di Raimondo, Caterina Lanna, Laura Diluvio, Sara Mazzilli, Virginia Garofalo, Emi Dika, Elena Dellambra, Filadelfo Coniglione, Luca Bianchi, Elena Campione

**Affiliations:** 1 Dermatology Unit, Department of Internal Medicine, Tor Vergata University, 00133 Rome, Italy; flavia.lozzi@hotmail.com (F.L.); cosimodiraimondo@gmail.com (C.D.R.); caterinalanna.cl@gmail.com (C.L.); lauradiluvio@yahoo.it (L.D.); saramazzilli2@gmail.com (S.M.); virginiagarofalo27@gmail.com (V.G.); 2 Dermatology Unit, Department of Experimental, Diagnostic and Specialty Medicine-DIMES, University of Bologna, Via Massarenti, 1-40138 Bologna, Italy; emi.dika@outlook.it; 3 Laboratory of Molecular and Cell Biology, Istituto Dermopatico dell’Immacolata–Istituto di Ricovero e Cura a Carattere Scientifico (IDI-IRCCS), via dei Monti di Creta 104, 00167 Rome, Italy; e.dellambra@idi.it; 4 Department of Clinical Science and Translational Medicine, Tor Vergata University, 00133 Rome, Italy; filadelfo.coniglione@uniroma2.it

**Keywords:** photosensitive drug, photosensitive drug-induced cutaneous eruption, phototoxic reaction, photoallergic reaction

## Abstract

Photosensitivity induced by drugs is a widely experienced problem, concerning both molecule design and clinical practice. Indeed, photo-induced cutaneous eruptions represent one of the most common drug adverse events and are frequently an important issue to consider in the therapeutic management of patients. Phototoxicity and photoallergy are the two different pathogenic mechanisms involved in photosensitization. Related cutaneous manifestations are heterogeneous, depending on the culprit drug and subject susceptibility. Here we report an updated review of the literature with respect to pathogenic mechanisms of photosensitivity, clinical manifestations, patient management, and prediction and evaluation of drug-induced photosensitivity. We present and discuss principal groups of photosensitizing drugs (antimicrobials, nonsteroidal anti-inflammatory drugs, anti-hypertensives, anti-arrhythmics, cholesterol, and glycemia-lowering agents, psychotropic drugs, chemotherapeutics, etc.) and their main damage mechanisms according to recent evidence. The link between the drug and the cutaneous manifestation is not always clear; more investigations would be helpful to better predict drug photosensitizing potential, prevent and manage cutaneous adverse events and find the most appropriate alternative therapeutic strategy.

## 1. Introduction

Photosensitive drugs (PSDs) can cause adverse cutaneous manifestations due to an interaction between the drugs themselves-either topically or systemically administered-and sun radiation [1]. UVA radiation (320–400 nm) is the main responsible for drug-induced eruptions, due to its deep penetration through the skin. UVB (290–320 nm) and visible light (400–700 nm) may also be involved, although less frequently [2,3].

Cutaneous eruptions represent one of the most common cutaneous adverse events (AEs) of PSDs (up to 8%) [3]. The prevalence of drug-induced photoreaction varies widely according to ethnic group and geographic area, probably due to different skin types, sunscreen use, and sun radiation intensity. In this regard, the percentage of patients with positive photopatch testing ranges between 5.7 and 49.5% depending on the country [4].

The pathogenesis of drug-induced photosensitivity includes phototoxic and photoallergic reactions, depending on the specific drug and the subject’s susceptibility. However, the distinction between the two pathogenetic mechanisms is often difficult. Clinical history, physical examination, diagnostic tests, and histopathologic features are the key points for diagnostic orientation [2,5].

Clinical cutaneous manifestations are heterogeneous and vary from itching on sun-exposed areas to severe blistering sunburn-like reactions [1]. More often, cutaneous manifestations present features compatible with those of contact dermatitis, showing eczematous eruption. In some instances, to reverse the cutaneous AEs it is sufficient to interrupt drug assumption, while in other cases it is necessary to start a topical or systemic therapy, usually based on corticosteroids or, in few cases, on immunosuppressant medications. Prevention consists of high sunscreen protection and the use of filtering clothing [2,4,5,6].

Some drugs are not only responsible for photosensitivity, but also an increased risk of cutaneous malignancies, such as melanoma and non-melanoma skin cancer (NMSC) [3,7,8,9].

Among the most important PSDs, there are antimicrobials, nonsteroidal anti-inflammatory drugs (NSAIDs), anti-hypertensives, anti-arrhythmic medications, psychotropic medications, chemotherapeutic agents, and others.

ICH Guidance S10 on Photosafety Evaluation of Pharmaceuticals 5 September 2015 was drawn up to establish international standards for photosafety assessment and harmonize these assessments, supporting human clinical trials and marketing authorizations for pharmaceutical products [10].

In this review, we discuss past knowledge and recent updates regarding the pathogenetic mechanisms of photosensitization, principal clinical manifestations of related cutaneous eruptions, and principal PSDs with their formulations, biochemical properties, and cutaneous AEs.

## 2. Pathogenesis

PSDs are exogenous chromophores, which absorb photons and undergo chemical reactions after exposure to a light source. The wavelengths absorbed depend on the chemical structure of the PSD [11].

As already mentioned, phototoxicity and photoallergy are the two mechanisms involved in PSD-induced reactions. Phototoxicity is more frequent and can occur in any individual exposed to a sufficient amount of the causative drug and light source. Differently, photoallergy occurs only in some individuals and requires an initial sensitization to the offending agent [12]. Table 1 summarizes the principal aspects of phototoxicity and photoallergy.

### 2.1. Phototoxicity

A photosensitizer chemical absorbs radiation energy and consequently rises to an excited state molecule. This process leads to oxygen-dependent reactions, which are ultimately responsible for cell damage through two main mechanisms [5].

The first one consists of the transfer of an electron to the excited-state photosensitizer and thus in the formation of free radicals that can react with biomolecules (directly or in the presence of oxygen), forming secondary free radicals (peroxyl radicals or hydroxyl radical). In the second type of reaction, the transfer of energy to ground-state oxygen causes oxygen radical formation. The consequent oxidation of lipids and proteins and DNA damage lead to cell injury [5,13,14]. Additional mechanisms include the formation of stable photoproducts responsible for tissue damage, and the covalent bondage of some photoproducts to target biological substrates (as for 8-methoxypsoralen and pyrimidine bases of DNA) [1,5,15]. The high variability of phototoxic manifestations can be partially related to the different sites of drug accumulation in the skin [1].

Cells have protective mechanisms against reactive oxygen species (ROS), such as the enzyme superoxide dismutase, glutathione peroxidase, and catalase. The damage occurs when these protective systems are overwhelmed, and when there are irreparable DNA alterations. This makes cells undergo apoptosis [1].

### 2.2. Photoallergy

Photoallergy was first reported in the early 1960s referred to as the use of halogenated salicylalinide and consists of a delayed immune response to photosensitizer-bound proteins [14].

Sensitization can occur through two possible models. The first one consists of photo-modification of the hapten (prohapten) that subsequently binds a protein and may potentially induce photoallergy. The second model first involves hapten-protein binding followed by light-mediated activation (photohapten). Either way, haptens undergo processing by Langerhans cells and are presented to naıve T cells in the lymph nodes, with consequent differentiation of photoallergy-specific T cells. The photoallergic reaction occurs when the patient is re-exposed to the allergen as well as to the appropriate radiation source and dose [4,12]. In this contest, there is the release of cytokines and chemokines and the recruitment of inflammatory cells with consequent eczematous eruption [1].

Immediate hypersensitivity has also been described in some cases of photoallergic reactions, due to an IgE response to UV [14,16,17].

## 3. Clinical Manifestations and Diagnostic Approach

Cutaneous manifestations induced by PSDs are usually limited to sun-exposed areas, although in some photoallergy reactions the lesions can spread over the entire skin surface [11]. Naturally shaded areas, such as the philtrum, submandibular and postauricular regions, and inverse areas, may be spared [4]. Clinical presentation is heterogeneous. Phototoxic reactions usually occur a few minutes to hours after light exposure and appear as an exaggerated sunburn, with erythema and edema together with itching and burning [1,11]. This scenario corresponds to keratinocyte direct damage, histologically visible as cell necrosis, and neutrophilic and lymphocytic infiltration of derma [2,11]. Photoallergic skin lesions appear 24 h or more after exposure to light and resemble eczematous dermatitis. Epidermal spongiosis, vesiculation, exocytosis of lymphocytes into the epidermis, and perivascular inflammatory infiltrates can be evidenced in the histologic examination. In a few cases, despite drug interruption, chronic actinic dermatitis may develop [11,18].

Other PSD-induced manifestations can be more characteristic. Pigmentation is frequent in phototoxic eruptions. Indeed, also a normal UV-response involves the release of IL-1α that stimulates the production of melanotrophins by keratinocytes. In addition, it has been shown that stress can influence the level of pro-inflammatory cytokines such as IL-1α [1,19]. Hyperpigmentation seems not to occur in photoallergic reactions [14,20]. Porphyria and pseudoporphyria are possible drug-induced manifestations. Porphyria is very rare and has been described as a consequence of methandrostenolone assumption [1,21]. Pseudoporphyria has cutaneous features similar to those of porphyria cutanea tarda with a normal porphyrin profile. Skin fragility leading to blisters is typical. Milia, hypertrichosis, and hyperpigmentation are rare [1]. Several drugs have been associated with drug-induced pellagra. Probably culprit drugs interfere with the metabolism of niacin/nicotinamide adenine dinucleotide (NAD) by inhibiting niacin production from dietary tryptophan and by acting as NAD analogs [1,22]. Erythema multiforme-like reactions and telangiectasia have also been described [4,23].

Two procedures are currently used to evaluate drug-induced photosensitivity, namely phototesting and photopatch testing. The first one aims at establishing the minimal erythema dose (MED) for a patient while taking a given medication and then after drug discontinuation. A lower MED during treatment suggests phototoxicity induced by the drug. The photopatch testing consists of applying two sets of a given medication onto the patient’s back. After 24 h, one set is irradiated with a dose of UVA below the MED. After twenty-four hours, the areas are examined for an eczematous eruption. A reaction present only at the irradiated site suggests a photoallergic reaction. The equal reaction at both sites expresses an allergic contact dermatitis. Lastly, a reaction on both the irradiated and non-irradiated area, but greater on the first one, suggests both allergic contact dermatitis and photoallergic reaction. Photopatch testing, however, has not been validated for systemic medications [3,4,24].

## 4. Prediction and Evaluation of Drug-Induced Photosensitivity

While developing a new drug it is important to assess its potential photosensitive effects. ROS assay is used to evaluate the photoreactivity of chemicals, consisting of the detection of reactive species produced after the irradiation of a chemical product. Additional tests are the photo-basophil-histamine-release test–which evaluates the release of inflammatory molecules from leucocytes-and the photo-hemolysis test, made to assess the damage to the cell membrane of the erythrocyte. The change in oxygen consumption in *Bacillus subtilis* and growth inhibition of yeast cells after irradiation are also used [1,25,26,27,28]. Culture models and human reconstituted epidermis may be useful to predict drug-induced photosensitivity [1]. UV spectral analysis is used to study the photoexcitability of chemicals and, together with the ROS assay, it is a validated analysis and thereby recommended in the ICH S10 guideline [10,14]. In addition, the photogenotoxic potential of a drug can be determined by establishing its affinity for DNA and its capability to induce structural alterations [1]. Other tests and molecular studies have been validated to assess the potential photosensitizing properties of drugs; however, their discussion is beyond the scope of this review.

## 5. Photosensitive Drugs

Here we report an updated analysis of the literature regarding the main drugs responsible for phototoxic and photoallergic reactions and the principal cutaneous manifestations associated with their administration. We focused on the principal culprit drugs and described the most characteristic reactions and the postulated damage mechanisms. We did not extend the discussion on every PSDs reported in the literature because it would have been beyond the scope of the review. The distinction between systemic drugs and topical formulations was considered the most appropriate.

### 5.1. Systemic Drugs

#### 5.1.1. Antimicrobials

Tetracyclines (tetracycline, doxycycline, minocycline, lymecycline) are the most frequent antimicrobials responsible for photosensitive reactions due to their wide absorption spectrum across the UVA region. Free radical activity during photodegradation of these drugs has also been reported [18]. Cutaneous reactions range from mild sunburn-like manifestations to widespread erythematous dermatitis. Solar urticarial, lichenoid reactions, actinic granuloma, and photo-induced onycholysis have also been described [3,29,30,31,32]. Interestingly, photo-induced onycholysis may appear even up to 2 weeks after UV exposure [33]. Recently, the carcinogenic risk due to the use of tetracycline has also been assessed, evidencing an 11% increase in the risk of developing basal cell carcinoma [34].

Nalidixic acid and fluoroquinolones are also responsible for several photo-induced eruptions with a broad spectrum of cutaneous manifestations (blistering, fragile skin, pseudoporphyria, and purpura) depending on the specific member of this group of agents [3]. The greatest phototoxic potential seems to be related to the halogen group in position 8. Members with a hydrogen group at this position show only mild phototoxic potential and those with a methoxy group in the same position appear to be the most photostable [3,35,36]. It has also been hypothesized that the photodefluorination leads to the production of a highly reactive radical capable of attacking cell biological constituents [18].

Cefotaxime and ceftazidime have been associated with photo-induced telangiectasia and sunburn susceptibility, respectively [3,37,38]. Photosensitive effects have also been reported for dapsone, trimethoprim, isoniazid, and pyrazinamide [39,40,41,42].

Among antifungal agents, voriconazole has been widely associated with severe photodrug-induced eruptions, and recent literature reported voriconazole as the second most common culprit of phototoxic reactions [3,43]. Cheilitis, pseudoporphyria, onycholysis, photoaging, and the development of melanoma and squamous cell carcinoma (SCC) on areas that have been affected by drug-induced eruption have also been reported [3,9,44,45,46].

Antimalarials may frequently cause photosensitization. Quinine and quinidine have been associated with edematous, eczematous, and lichenoid eruptions, while chloroquine and hydroxychloroquine have been associated with polymorphous light eruption and systemic lupus erythematosus [3,47,48,49,50,51]. Recently photo-induced eruptions have been also described in patients assuming atovaquone/proguanil [52].

Among antiretrovirals, a photosensitive potential has been reported for efavirenz, tenofovir, and tipranavir. However, in the context of HIV infections, it is not always simple to distinguish cutaneous manifestations related to the disease itself from those induced by the drug [3,53,54,55]. Indeed, nearly 5% of patients with HIV suffer from some form of photosensitive dermatitis, including PSDs-induced reactions, actinic prurigo, chronic actinic dermatitis, lichenoid photoeruptions, porphyria cutanea tarda, pseudoporphyria, photoaggravated granuloma annulare, and actinic lichenoid leukomelanoderma [56]. Moreover, HIV patients are overall more susceptible to drug reactions than the general population, due to multiple factors among which there are polypharmacy, slow acetylator status, relative glutathione deficiency, and latent Herpesviridae infections [55].

#### 5.1.2. Nonsteroidal Anti-Inflammatory Drugs (NSAIDs)

NSAIDs include many molecules with a different chemical structure but consistent in their ability to inhibit the cyclo-oxygenase (COX) enzymes to varying degrees. The old NSAIDs have their target in the COX-1 enzyme, whereas the most recent NSAIDs inhibit preferentially the COX-2 enzyme [18]. Cutaneous manifestations range from eczematous lesions to erythema multiforme to lichenoid eruptions [4].

It has been evidenced that the most photoactive NSAIDs are the 2-arylpropionic acid derivatives (naproxen, ibuprofen, ketoprofen, suprofen, benoxaprofen, and tiaprofenic acid) which lead to the generation of significant yields of singlet oxygen and free radical species upon UV irradiation [18,57,58]. Naproxen appears to have the greatest photosensitizing potential and has even been associated with pseudoporphyria [3,59,60]. It has been proposed that the different schedules of administration of NSAIDs can partly explain the different photosensitizing potential of these drugs. Indeed, naproxen is administered at a higher dosage than indomethacin or diclofenac, thus it has a higher circulating concentration compared to the latter. Among the most recent NSAIDs, photoallergic reactions and pseudoporphyria have been reported in relation to celecoxib assumption although data are not yet sufficient [61].

#### 5.1.3. Anti-Hypertensives

The three main categories of anti-hypertensives most frequently involved in photo-induced cutaneous reactions are diuretics, ACE inhibitors, and angiotensin receptor blockers (ARBs).

Thiazides are the most commonly prescribed diuretics. Hydrochlorothiazide has been associated with exaggerated sunburn reactions, eczematous and lichenoid eruptions, and photoleukomelanoderma [62,63]. It is interesting to note that in some cases chronic eczematous photosensitivity has been described as lasting months to years after discontinuation of the drug [64]. As previously mentioned, although less frequently, UVB can also be responsible for drug photo-induced cutaneous reactions. Hydrochlorothiazide has been recently associated with the phototoxic reaction after UVB exposure [65]. Furosemide, another diuretic, has also been reported to cause phototoxic eruptions typically bullous lesions resembling Brunsting–Perry type bullous pemphigoid [66,67]. Hydrochlorothiazide and furosemide have in common an aromatic chlorine substituent in their molecular structure. This characteristic has been postulated to be responsible for the photosensitizing properties of these drugs linked to an intermediate photoionisation process and bond dissociation occurring during the irradiation, which in turn is responsible for the production of free radicals, followed then by cell damage. Indeed, all chlorine-containing drugs that undergo photodechlorination have been included in the list of PSDs [18]. As for ACE inhibitors, ramipril, quinapril, and enalapril have been reported to cause photosensitivity. Fewer data are available for ARBs, although photo-induced cutaneous reactions have been associated with losartan, irbesartan, and valsartan, and up to 10% have been reported as serious [3,68,69,70]. Calcium channel blockers (CCBs) belonging to the dihydropyridine group-which includes amlodipine and nifedipine-have been associated with photodistributed facial telangiectasia and photodermatitis. Diltiazem has caused in some cases photodistributed hyperpigmentation [3,71,72,73].

It is important to note that recent evidence has linked hydrochlorothiazide assumption to an increased risk of developing NMSCs, partly as a consequence of its photosensitizing effect [74,75]. In this regard, topical 0.8% piroxicam and 50+ sunscreen filters proved to be highly effective in the treatment of actinic keratoses in patients assuming anti-hypertensives, especially photosensitizing thiazides. Similar results have been reported for ingenol mebutate [76,77].

A recent study has also reported an increased risk of both SCC and melanoma in patients taking amiloride and hydrochlorothiazide, and an increased risk of melanoma in patients taking indapamide [3,8]. In addition, a meta-analysis has highlighted an increased risk of skin cancer and cutaneous melanoma respectively in patients taking CCBs and β-blockers [78]. Recently, beta-blockers, whose therapeutic indications include hypertension as well as arrhythmias, heart failure, and essential tremor, have been associated with photosensitization. Moreover, it increases their consideration as a potentially effective treatment in dermatologic diseases as hemangiomas, wound healing, Kaposi sarcoma, melanoma, pyogenic granuloma, and pemphigus. For this reason, it would be important to acquire more data about their cutaneous AEs [79]. Tilisolol, bisoprolol, and atenolol have been mainly associated with photo-induced cutaneous reactions [3,80].

#### 5.1.4. Anti-Arrhythmics

Amiodarone has been associated with several AEs, including photosensitivity, in a wide number of patients. In particular, phototoxicity has been reported in nearly 7% of patients taking amiodarone [81]. Photo-induced cutaneous manifestations typically occur as a burning sensation followed by erythema and eczematous eruption. Usually, after long-term exposure, amiodarone induces a distinctive blue-grey pigmentation on sun-exposed areas in 1–2% of patients [81,82,83]. Amiodarone, and its principal metabolite desethylamiodarone, accumulate in the skin, where they can be detected in pigmented areas at a concentration ten times greater than that detectable in non-pigmented areas. Moreover, it has been demonstrated that iodine-rich amiodarone and its metabolite store in secondary lysosomes bounded to lipofuscin, as a consequence of dermal macrophages phagocytosis. This explains typical long-lasting (1–2 years) skin pigmentation [3,84]. Dronedarone shows significantly minor phototoxicity than amiodarone, although cases of photo-induced reactions have been described [85].

#### 5.1.5. Chemotherapeutics

Several groups of chemotherapeutics have been associated with photosensitivity reactions. Among the antimetabolites, fluorouracil has been linked to enhanced sunburn reactions, photodistributed hyperpigmentation, and polymorphous light eruption-like reactions [86]. Tegafur, capecitabine, and dacarbazine also cause photo-induced lichenoid and eczematous reactions [3]. As for antimitotic agents, taxanes have been widely associated with photosensitization. Paclitaxel has been reported to cause both photodistributed erythema multiforme and onycholysis [87,88]. Nanoparticle albumin-bound paclitaxel derivative (nab-paclitaxel) has been recently associated with photosensitivity reactions [89].

Targeted therapies-nowadays a topic of increasing relevance–may also show AEs, including photosensitivity. Vemurafenib is one of the most common culprits associated with photosensitivity reactions, which have been observed in 35–63% of patients treated with this drug. Noteworthy, the photosensitizing activity of vemurafenib does not appear to be involved in the increased risk of developing SCC described in patients taking this drug [90,91].

New attention is now being given also to Janus kinase (JAK) inhibitors. A recent study reported a photodistributed rash in a patient with polycythemia vera occurring one month after starting the treatment with ruxolitinib. In addition, the patient developed multiple SCC [92].

**Figure 1:**
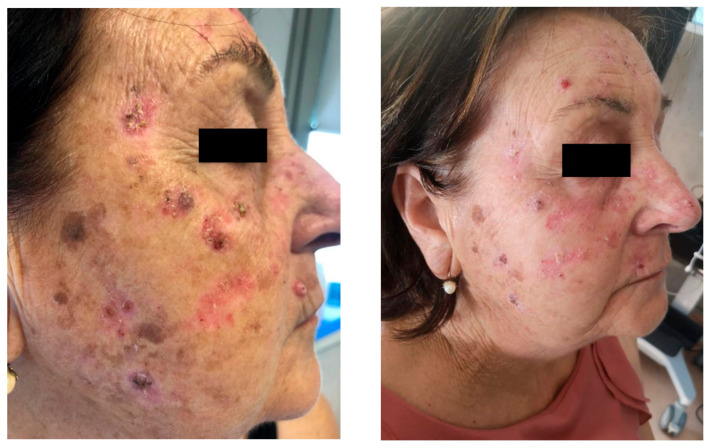
Photo-induced dermatitis in a patient assuming hydroxyurea.

Hydroxyurea has been associated with photo-induced dermatitis and granulomatous eruption [93,94] (Figure 1).

#### 5.1.6. Psychotropic Drugs

Chlorpromazine is the prototype of PSDs. Its chemical structure is characterized by the presence of an aromatic chlorine substituent, sharing with hydrochlorothiazide and furosemide the postulated pathogenic mechanisms of photosensitization described in Section 5.1.3 [18]. It has been associated with exaggerated sunburn reactions, lichenoid reactions, and bullous eruptions. Photodistributed slate-grey to violaceous hyperpigmentation has been reported in the case of long-term exposure to chlorpromazine or thioridazine [95].

The tricyclic antidepressants chemically related to phenothiazines have also shown photosensitizing effects with a broad spectrum of cutaneous manifestations, ranging from erythematous eruptions, blistering, blue-grey hyperpigmentation, photodistributed granuloma annulare, telangiectasia [3,4].

Phenothiazines also share some structural features with ethylenediamine-derived antihistamines, such as cetirizine and hydroxyzine. This may lead to possible cross-reactions [4].

#### 5.1.7. Others

Among the HMG-CoA reductase inhibitors (statins), simvastatin and atorvastatin have been reported to cause photodistributed erythema multiforme [96]. Atorvastatin phototoxicity seems to be due to singlet oxygen generation via a phenanthrene-like photoproduct [97]. Fenofibrate has been linked to eczematous and lichenoid eruptions [98].

Diabetic medications have been shown to cause photo-induced cutaneous lesions. In particular, metformin has been associated with both erythematous and eczematous eruptions [99].

Clopidogrel, an antiplatelet drug, has been associated with a lichenoid photodistributed eruption [100].

Table 2 lists discussed drugs.

### 5.2. Topical Drugs and Cosmetics

Topical agents can be associated with both phototoxic and photoallergic reactions. It has been reported that acyclovir, dibucaine injection, hydrocortisone, and chlorpromazine gel may cause photoallergic reactions [11,101,102,103,104].

Furocoumarins, such as bergapten, 5- and 8-methoxypsoralen, are photosensitive compounds produced by plants and used in the cosmetic industry and folk medicine. They have been associated with phototoxic reactions and phytophotodermatitis, often presenting bullous dermatitis with hyperpigmentation [11]. Coal tar has also been identified as a phototoxic reaction inducer, causing an immediate burning sensation, followed by wheals and then by erythema within 24–48 h [11,105].

NSAIDs are among the topical agents most frequently responsible for photosensitization. Ketoprofen can cause both phototoxic and photoallergic reactions-the latter ones more frequently. Dermatitis is often characterized by edema, bullae, or erythema multiforme that can also extend beyond the area of application and persist after drug discontinuation [11,106]. Benzophenone plays a major role in ketoprofen photosensitive effects [107] and cross-reactions have been described for tiaprofenic acid, suprofen, oxybenzone, and fenofibrate [11,107,108]. A cross-reactivity in photoallergic sensitization has also been described between diclofenac and aceclofenac, both responsible for vesicular dermatitis [11,107,109,110].

The topical application of benzydamine has been associated with photoallergic reactions locally and at distant sites as well as with submental dermatitis and cheilitis when used orally [11,111,112]. Piroxicam may even induce photoallergic reactions which appear usually on the face and dorsum of the hands as erythematous papules, vesicles, and dyshidrosis. Patients most frequently reported previous sensitization with thimerosal [11,113,114].

Interestingly, although systemic tetracyclines have been widely associated with photosensitizing potential, a recent study reported no evidence of phototoxicity, photoallergy, skin sensitization, or skin irritation with topical minocycline foam 4% [115].

Cosmetics ingredients often show photoallergenic and photoirritance potential. This is partially because cosmetics substances are formulated to remain on the skin despite rinsing, thus increasing the risk of toxic reactions with repeated applications [14]. 6-methylcoumarin, musk ambrette, and hexachlorophene have been associated with numerous cutaneous eruptions and their use has been stopped [116]. To date, most photoallergic reactions are caused by products, often sunscreens, containing *p*-aminobenzoic acid (PABA) derivatives, benzophenones, salicylates, dibenzoylmethane derivatives, and anthranilates [14,117]. In addition, some derivatives of benzophenones show structural similarities with ketoprofen, leading often to cross-reactivity [118]. It should be noted that newer sunscreens contain molecules more photostable, such as Mexoryl SX-for which only one case of photoallergy has been reported-Tinosorb S and M [11,119]. Many antibacterials have also been associated with photosensitive effects, including triclosan (soap and deodorants) and fenticlor (hair care products), characterized by the low and moderate potential of photoallergy [14,116,120]. Table 3 lists the discussed topical drugs and cosmetics.

## 6. Discussion

Cutaneous eruptions induced by drugs are frequently experienced during clinical practice and drug-related photosensitivity, specifically, may represent an important issue to consider in the therapeutic management of patients. The PSD list is being updated constantly because of increasing therapeutic strategies and clinical experience. The knowledge of the pathogenetic mechanisms of drug-induced photosensitivity appears to be an important clue for both the prediction of the photosensitizing potential of a drug according to its chemical structure and the prevention of cutaneous damage. Phototoxicity mostly relies on oxygen-dependent reactions, which lead to oxidation and damage of important cellular structures, including DNA. As discussed above, it has been demonstrated that some PSDs are responsible not only for cutaneous eruptions but also for an increased risk of cutaneous carcinomas and melanoma. In this regard, prevention appears to be essential. It has been evidenced that vitamin D3 has a protective effect on keratinocytes affected by oxidative DNA damage as well as by UVB damage [1,121,122]. Moreover, it has been shown that the nuclear erythroid 2-related factor 2 (Nrf2) is important in the induction of some drug-metabolizing enzymes, such as glutathione S-transferase and NAD(P)H:quinone oxidoreductase 1 [123]. The induction of Nrf2 is being explored as a therapeutic option to protect skin cells against UV-induced oxidative stress and photosensitivity [1,124,125].

Clinical manifestations are extremely heterogeneous, depending on the drug and patient susceptibility. Some eruptions are more indicative than others but accurate medical history is essential and diagnostic tests and histopathologic examination are frequently necessary, also considering that patients often assume more than one drug. In this regard, it is reasonable to think that patients taking multiple drugs have a greater risk to be affected by photo-induced cutaneous reactions, both for the potential accumulation of phototoxic effects and possible cross-reactivity between drugs with regard to photoallergic reactions. Differential diagnosis with photorecall reactions should be considered. Photorecall reaction consists of a sunburn-like eruption appearing after drug administration in the absence of the radiation trigger, in the same areas affected by a previously sunburn. This pathological event has been most commonly described in association with chemotherapeutic agents, such as methotrexate, docetaxel, and gemcitabine and it should be distinguished from true drug-induced photosensitivity [3,126,127,128]. Although less frequently, antibiotics can also be responsible for this clinical manifestation [129].

As previously reported, some PSDs are responsible for drug-induced pellagra probably due to interference with NAD metabolism. The fact that NAD is the main substrate of Poly (ADP-ribose) polymerase 1 (PARP1)-an enzyme involved in repairing UV-induced DNA damage–may explain this pathologic manifestation [1,22]. Overactivation of PARP by excessive UV exposure can in turn lead to cellular NAD and ATP depletion with consequent cellular energy impairment and cell death [130]. In this regard, the role of nicotinamide has been investigated, the primary precursor of NAD and a PARP inhibitor. It has been evidenced that nicotinamide reduces UV-induced inflammation, cellular energy depletion, and immunosuppression, and enhances the repair of UV-induced DNA damage [130,131,132]. This makes nicotinamide an important option for photoprotection and skin cancer chemoprevention and it deserves further investigation to better define its role in PSD-induced phototoxicity and photoallergy.

## 7. Conclusions

In conclusion, PSDs represent an important research area and more investigations would be helpful to better predict drug photosensitizing potential, prevent and manage cutaneous adverse events and find the most appropriate alternative therapeutic strategy.

## Figures and Tables

**Table 1:** Main features of phototoxicity and photoallergy.

Characteristics	Phototoxicity	Photoallergy
Incidence	high	Low
Sensitization	+	−
Time of onset	Few min-h	>24 h
Most common clinical presentation	Exaggerate sunburn	Dermatitis
Dose dependency	+	+/−
Histologic features	cell necrosis and, neutrophilic and lymphocytic infiltration of derma	Epidermal spongiosis, vesiculation, exocytosis of lymphocytes into the epidermis, perivascular inflammatory infiltrates

min: minutes, h: hours.

**Table 2:** Main groups and relatively molecules of photosensitive drugs discussed/cited in the text.

PSD Group	Molecule
Antimicrobials	Tetracycline Doxycycline Minocycline Lymecycline Nalidix acid and fluorochinolones Cefotaxime Ceftazidime Dapsone Trimethoprim Isoniazid Pyrazinamide Voriconazole Quinine Quinidine Chloroquine Hydroxychloroquine Atovaquone/proguanil Efavirenz Tenofovir Tipranavir
NSAIDs	Naproxen Ibuprofen Ketoprofen Suprofen Benoxaprofen Tiaprofenic acid
Antihypertensives	Hydrochlorothiazide Furosemide Ramipril Quinapril Enalapril
Antihypertensives	Losartan Irbesartan Valsartan Amlodipine Nifedipine Diltiazem Amiloride Indapamide Tilisolol Bisoprolol Atenolol
Antiarrhythmics	Amiodarone Dronedarone
Chemotherapeutics	Fluorouracil Tegafur Capecitabine Dacarbazine Paclitaxel and nab-paclitaxel Vemurafenib Ruxolitinib Hydroxyurea
Psychotropic drugs	Chlorpromazine Thioridazine Tricyclic antidepressant
Others	Simvastatin, atorvastatin, fenofibrate, metformin, clopidogrel

PSD: photosensitive drug; NSAID: nonsteroidal anti-inflammatory drugs.

**Table 3:** Topical drugs and cosmetics discussed/cited in the text.

Topical Drugs	Cosmetics
Acyclovir Dibucaine Hydrocortisone Chlorpromazine gel Furocoumarins (bergapten, 5- and 8-methoxypsoralen) Coal tar Ketoprofen Tiaprofenic acid Suprofen Oxybenzone Fenofibrate Diclofenac Aceclofenac Benzydamine Piroxicam	Furocoumarins (bergapten, 5- and 8-methoxypsoralen) 6-methylcoumarin Musk ambrette Hexachlorophene PABA derivatives Benzophenones Salicylates Dibenzoylmethane derivatives Anthranilates Mexoryl SX Triclosan Fenticlor

PABA: *p*-aminobenzoic acid.

## References

[1] Khandpur S., Porter R.M., Boulton S.J., Anstey A. (2017). Drug-induced photosensitivity: New insights into pathomechanisms and clinical variation through basic and applied science. Br. J. Dermatol..

[2] Drucker A.M., Rosen C.F. (2011). Drug-induced photosensitivity: Culprit drugs, management and prevention. Drug Saf..

[3] Blakely K.M., Drucker A.M., Rosen C.F. (2019). Drug-induced photosensitivity—An update: Culprit drugs, prevention and management. Drug Saf..

[4] Hinton A.N., Goldminz A.M. (2020). Feeling the burn: Phototoxicity and photoallergy. Dermatol. Clin..

[5] Kutlubay Z., Sevim A., Engin B., Tuzun Y. (2014). Photodermatoses, including phototoxic and photoallergic reactions (internal and external). Clin. Dermatol..

[6] Wilm A., Berneburg M. (2015). Photoallergy. J. Dtsch. Dermatol. Ges..

[7] Addo H.A., Ferguson J., Frain-Bell W. (1987). Thiazide-induced photosensitivity: A study of 33 subjects. Br. J. Dermatol..

[8] Jensen A.O., Thomsen H.F., Engebjerg M.C., Olesen A.B., Sorensen H.T., Karagas M.R. (2008). Use of photosensitising diuretics and risk of skin cancer: A population-based case-control study. Br. J. Cancer.

[9] Williams K., Mansh M., Chin-Hong P., Singer J., Arron S.T. (2014). Voriconazole-associated cutaneous malignancy: A literature review on photocarcinogenesis in organ transplant recipients. Clin. Infect. Dis..

[10] Food and Drug Administration, HHS (2015). International Conference on Harmonisation; S10 Photosafety Evaluation of Pharmaceuticals; guidance for industry; availability. Notice. Fed. Regist..

[11] Monteiro A.F., Rato M., Martins C. (2016). Drug-induced photosensitivity: Photoallergic and phototoxic reactions. Clin. Dermatol..

[12] Andreu I., Mayorga C., Miranda M.A. (2010). Generation of reactive intermediates in photoallergic dermatitis. Curr. Opin. Allergy Clin. Immunol..

[13] Lehmann P. (2006). Diagnostic approach to photodermatoses. J. Dtsch. Dermatol. Ges..

[14] Onoue S., Seto Y., Sato H., Nishida H., Hirota M., Ashikaga T., Api A.M., Basketter D., Tokura Y. (2017). Chemical photoallergy: Photobiochemical mechanisms, classification, and risk assessments. J. Dermatol. Sci..

[15] Cimino G.D., Gamper H.B., Isaacs S.T., Hearst J.E. (1985). Psoralens as photoactive probes of nucleic acid structure and function: Organic chemistry, photochemistry, and biochemistry. Ann. Rev. Biochem..

[16] Vassileva S.G., Mateev G., Parish L.C. (1998). Antimicrobial photosensitive reactions. Arch. Intern. Med..

[17] Storck H. (1965). Photoallergy and photosensitivity due to systemically administered drugs. Arch. Dermatol..

[18] Moore D.E. (2002). Drug-induced cutaneous photosensitivity: Incidence, mechanism, prevention and management. Drug Saf..

[19] Inoue K., Hosoi J., Ideta R., Ohta N., Ifuku O., Tsuchiya T. (2003). Stress augmented ultraviolet-irradiation-induced pigmentation. J. Investig. Dermatol..

[20] Deleo V.A. (2004). Photocontact dermatitis. Dermatol. Ther..

[21] Lane P.R., Massey K.L., Worobetz L.J., Jutras M.N., Hull P.R. (1994). Acute hereditary coproporphyria induced by the androgenic/anabolic steroid methandrostenolone (Dianabol). J. Am. Acad. Dermatol..

[22] Benavente C.A., Schnell S.A., Jacobson E.L. (2012). Effects of niacin restriction on sirtuin and PARP responses to photodamage in human skin. PLoS ONE.

[23] Collins P., Ferguson J. (1993). Photodistributed nifedipine-induced facial telangiectasia. Br. J. Dermatol..

[24] Kerr A., Shareef M., Dawe R., Ferguson J. (2010). Photopatch testing negative in systemic quinine phototoxicity. Photodermatol. Photoimmunol. Photomed..

[25] Onoue S., Yamauchi Y., Kojima T., Igarashi N., Tsuda Y. (2008). Analytical studies on photochemical behavior of phototoxic substances; effect of detergent additives on singlet oxygen generation. Pharm. Res..

[26] Beani J.C., Gautron R., Amblard P., Bastrenta F., Harrouch L., Jardon P., Reymond J.L. (1985). Screening for drug photosensitization activity by measuring the variations in oxygen consumption of Bacillus subtilis. Photo-Dermatol..

[27] Selvaag E. (1997). Evaluation of phototoxic properties of oral antidiabetics and diuretics. Photohemolysis model as a screening method for recognizing potential photosensitizing drugs. Arzneimittelforschung.

[28] Przybilla B., Schwab-Przybilla U., Ruzicka T., Ring J. (1987). Phototoxicity of non-steroidal anti-inflammatory drugs demonstrated in vitro by a photo-basophil-histamine-release test. Photo-Dermatol..

[29] Lim D.S., Triscott J. (2003). O’Brien’s actinic granuloma in association with prolonged doxycycline phototoxicity. Australas. J. Dermatol..

[30] Susong J., Carrizales S. (2014). Lichenoid photosensitivity: An unusual reaction to doxycycline and an unusual response. Cutis.

[31] Gventer M., Brunetti V.A. (1985). Photo-onycholysis secondary to tetracycline. A case report. J. Am. Podiatr. Med. Assoc..

[32] Badri T., Ben Tekaya N., Cherif F., Ben Osman Dhahri A. (2004). Photo-onycholysis: Two cases induced by doxycycline. Acta Dermatovenerol. Alp Pannonica Adriat.

[33] Rabar D., Combemale P., Peyron F. (2004). Doxycycline-induced photo-onycholysis. J. Travel Med..

[34] Li W.Q., Drucker A.M., Cho E., Laden F., VoPham T., Li S., Weinstock M.A., Qureshi A.A. (2018). Tetracycline use and risk of incident skin cancer: A prospective study. Br. J. Cancer.

[35] Sanchez J.P., Gogliotti R.D., Domagala J.M., Gracheck S.J., Huband M.D., Sesnie J.A., Cohen M.A., Shapiro M.A. (1995). The synthesis, structure-activity, and structure-side effect relationships of a series of 8-alkoxy- and 5-amino-8-alkoxyquinolone antibacterial agents. J. Med. Chem..

[36] Hayashi N., Nakata Y., Yazaki A. (2004). New findings on the structure-phototoxicity relationship and photostability of fluoroquinolones with various substituents at position 1. Antimicrob. Agents Chemother..

[37] Borgia F., Vaccaro M., Guarneri F., Cannavo S.P. (2000). Photodistributed telangiectasia following use of cefotaxime. Br. J. Dermatol..

[38] Vinks S.A., Heijerman H.G., de Jonge P., Bakker W. (1993). Photosensitivity due to ambulatory intravenous ceftazidime in cystic fibrosis patient. Lancet.

[39] De D., Dogra S., Kaur I. (2007). Dapsone induced acute photosensitivity dermatitis; a case report and review of literature. Lepr. Rev..

[40] Chandler M.J. (1986). Recurrence of phototoxic skin eruption due to trimethoprim. J. Infect. Dis..

[41] Lee A.Y., Jung S.Y. (1998). Two patients with isoniazid-induced photosensitive lichenoid eruptions confirmed by photopatch test. Photodermatol. Photoimmunol. Photomed..

[42] Katiyar S.K., Bihari S., Prakash S. (2010). Pyrazinamide-induced phototoxicity: A case report and review of literature. Indian J. Dermatol..

[43] Kim W.B., Shelley A.J., Novice K., Joo J., Lim H.W., Glassman S.J. (2018). Drug-induced phototoxicity: A systematic review. J. Am. Acad. Dermatol..

[44] Kolaitis N.A., Duffy E., Zhang A., Lo M., Barba D.T., Chen M., Soriano T., Hu J., Nabili V., Saggar R. (2017). Voriconazole increases the risk for cutaneous squamous cell carcinoma after lung transplantation. Transpl. Int..

[45] Miller D.D., Cowen E.W., Nguyen J.C., McCalmont T.H., Fox L.P. (2010). Melanoma associated with long-term voriconazole therapy: A new manifestation of chronic photosensitivity. Arch. Dermatol..

[46] Haylett A.K., Felton S., Denning D.W., Rhodes L.E. (2013). Voriconazole-induced photosensitivity: Photobiological assessment of a case series of 12 patients. Br. J. Dermatol..

[47] Gould J.W., Mercurio M.G., Elmets C.A. (1995). Cutaneous photosensitivity diseases induced by exogenous agents. J. Am. Acad. Dermatol..

[48] Ferguson J., Addo H.A., Johnson B.E., Frain-Bell W. (1987). Quinine induced photosensitivity: Clinical and experimental studies. Br. J. Dermatol..

[49] Ljunggren B., Hindsen M., Isaksson M. (1992). Systemic quinine photosensitivity with photoepicutaneous cross-reactivity to quinidine. Contact Dermat..

[50] van Weelden H., Bolling H.H., Baart de la Faille H., van der Leun J.C. (1982). Photosensitivity caused by chloroquine. Arch. Dermatol..

[51] Lisi P., Assalve D., Hansel K. (2004). Phototoxic and photoallergic dermatitis caused by hydroxychloroquine. Contact Dermat..

[52] Amelot A., Dupouy-Camet J., Jeanmougin M. (2014). Phototoxic reaction associated with Malarone (atovaquone/proguanil) antimalarial prophylaxis. J. Dermatol..

[53] Furue M. (2004). Photosensitive drug eruption induced by efavirenz in a patient with HIV infection. Intern. Med..

[54] Verma R., Vasudevan B., Shankar S., Pragasam V., Suwal B., Venugopal R. (2012). First reported case of tenofovir-induced photoallergic reaction. Indian J. Pharmacol..

[55] Hoosen K., Mosam A., Dlova N.C., Grayson W. (2019). An update on adverse cutaneous drug reactions in HIV/AIDS. Dermatopathology.

[56] Koch K. (2017). Photosensitive disorders in HIV. South Afr. J. HIV Med..

[57] Bosca F., Miranda M.A., Vano L., Vargas F. (1990). New photodegradation pathways for naproxen, a phototoxic nonsteroidal antiinflammatory drug. J. Photoch. Photobio. A.

[58] Costanzo L.L., De Guidi G., Condorelli G., Cambria A., Fama M. (1989). Molecular mechanism of drug photosensitization—II. Photohemolysis sensitized by ketoprofen. Photochem. Photobiol..

[59] LaDuca J.R., Bouman P.H., Gaspari A.A. (2002). Nonsteroidal antiinflammatory drug-induced pseudoporphyria: A case series. J. Cutan. Med. Surg..

[60] Al-Khenaizan S., Schechter J.F., Sasseville D. (1999). Pseudoporphyria induced by propionic acid derivatives. J. Cutan. Med. Surg..

[61] Yazici A.C., Baz K., Ikizoglu G., Kokturk A., Uzumlu H., Tataroglu C. (2004). Celecoxib-induced photoallergic drug eruption. Int. J. Dermatol..

[62] Gomez-Bernal S., Alvarez-Perez A., Rodriguez-Pazos L., Gutierrez-Gonzalez E., Rodriguez-Granados M.T., Toribio J. (2014). Photosensitivity due to thiazides. Actas Dermosifiliogr..

[63] Johnston G.A. (2002). Thiazide-induced lichenoid photosensitivity. Clin. Exp. Dermatol..

[64] Robinson H.N., Morison W.L., Hood A.F. (1985). Thiazide diuretic therapy and chronic photosensitivity. Arch. Dermatol..

[65] Rosenthal A., Herrmann J. (2019). Hydrochlorothiazide-induced photosensitivity in a psoriasis patient following exposure to narrow-band ultraviolet B excimer therapy. Photodermatol. Photoimmunol. Photomed..

[66] Burry J.N., Lawrence J.R. (1976). Phototoxic blisters from high frusemide dosage. Br. J. Dermatol..

[67] Takeichi S., Kubo Y., Arase S., Hashimoto T., Ansai S. (2009). Brunsting-Perry type localized bullous pemphigoid, possibly induced by furosemide administration and sun exposure. Eur. J. Dermatol..

[68] Rodriguez Granados M.T., Abalde T., Garcia Doval I., De la Torre C. (2004). Systemic photosensitivity to quinapril. J. Eur. Acad. Dermatol. Venereol..

[69] Wagner S.N., Welke F., Goos M. (2000). Occupational UVA-induced allergic photodermatitis in a welder due to hydrochlorothiazide and ramipril. Contact Dermat..

[70] Viola E., Coggiola Pittoni A., Drahos A., Moretti U., Conforti A. (2015). Photosensitivity with angiotensin II receptor blockers: A retrospective study using data from VigiBase^®^. Drug Saf..

[71] Bakkour W., Haylett A.K., Gibbs N.K., Chalmers R.J., Rhodes L.E. (2013). Photodistributed telangiectasia induced by calcium channel blockers: Case report and review of the literature. Photodermatol. Photoimmunol. Photomed..

[72] Boyer M., Katta R., Markus R. (2003). Diltiazem-induced photodistributed hyperpigmentation. Dermatol. Online J..

[73] Scherschun L., Lee M.W., Lim H.W. (2001). Diltiazem-associated photodistributed hyperpigmentation: A review of 4 cases. Arch. Dermatol..

[74] Garrido P.M., Borges-Costa J. (2020). Hydrochlorothiazide treatment and risk of non-melanoma skin cancer: Review of the literature. Rev. Port. Cardiol..

[75] (2019). MHRA drug safety update: Hydrochlorothiazide and non-melanoma skin cancer. Drug Ther. Bull..

[76] Mazzilli S., Garofalo V., Ventura A., Diluvio L., Milani M., Bianchi L., Campione E. (2018). Effects of topical 0.8% piroxicam and 50+ sunscreen filters on actinic keratosis in hypertensive patients treated with or without photosensitizing diuretic drugs: An observational cohort study. Clin. Cosmet. Investig. Dermatol..

[77] Campione E., Di Prete M., Diluvio L., Bianchi L., Orlandi A. (2016). Efficacy of ingenol mebutate gel for actinic keratosis in patients treated by thiazide diuretics. Clin. Cosmet. Investig. Dermatol..

[78] Gandini S., Palli D., Spadola G., Bendinelli B., Cocorocchio E., Stanganelli I., Miligi L., Masala G., Caini S. (2018). Anti-hypertensive drugs and skin cancer risk: A review of the literature and meta-analysis. Crit. Rev. Oncol. Hematol..

[79] Tatu A.L., Elisei A.M., Chioncel V., Miulescu M., Nwabudike L.C. (2019). Immunologic adverse reactions of beta-blockers and the skin. Exp. Ther. Med..

[80] Alrashidi A., Rhodes L.E., Sharif J.C.H., Kreeshan F.C., Farrar M.D., Ahad T. (2020). Systemic drug photosensitivity-Culprits, impact and investigation in 122 patients. Photodermatol. Photoimmunol. Photomed..

[81] Bongard V., Marc D., Philippe V., Jean-Louis M., Maryse L.M. (2006). Incidence rate of adverse drug reactions during long-term follow-up of patients newly treated with amiodarone. Am. J. Ther..

[82] Harris L., McKenna W.J., Rowland E., Holt D.W., Storey G.C., Krikler D.M. (1983). Side effects of long-term amiodarone therapy. Circulation.

[83] Rappersberger K., Honigsmann H., Ortel B., Tanew A., Konrad K., Wolff K. (1989). Photosensitivity and hyperpigmentation in amiodarone-treated patients: Incidence, time course, and recovery. J. Investig. Dermatol..

[84] Zachary C.B., Slater D.N., Holt D.W., Storey G.C., MacDonald D.M. (1984). The pathogenesis of amiodarone-induced pigmentation and photosensitivity. Br. J. Dermatol..

[85] Ladizinski B., Elpern D.J. (2013). Dronaderone-induced phototoxicity. J. Drugs Dermatol..

[86] Falkson G., Schulz E.J. (1962). Skin changes in patients treated with 5-fluorouracil. Br. J. Dermatol..

[87] Cohen P.R. (2009). Photodistributed erythema multiforme: Paclitaxel-related, photosensitive conditions in patients with cancer. J. Drugs Dermatol..

[88] Hussain S., Anderson D.N., Salvatti M.E., Adamson B., McManus M., Braverman A.S. (2000). Onycholysis as a complication of systemic chemotherapy: Report of five cases associated with prolonged weekly paclitaxel therapy and review of the literature. Cancer.

[89] Beutler B.D., Cohen P.R. (2015). Nab-paclitaxel-associated photosensitivity: Report in a woman with non-small cell lung cancer and review of taxane-related photodermatoses. Dermatol. Pract. Concept..

[90] Lacouture M.E., Duvic M., Hauschild A., Prieto V.G., Robert C., Schadendorf D., Kim C.C., McCormack C.J., Myskowski P.L., Spleiss O. (2013). Analysis of dermatologic events in vemurafenib-treated patients with melanoma. Oncologist.

[91] Gelot P., Dutartre H., Khammari A., Boisrobert A., Schmitt C., Deybach J.C., Nguyen J.M., Seite S., Dreno B. (2013). Vemurafenib: An unusual UVA-induced photosensitivity. Exp. Dermatol..

[92] Khanna U., Richardson V., Hexner E., Nguyen C.V., Elenitsas R., Rosenbach M. (2019). A photo-distributed papulopustular eruption and multiple squamous cell carcinomas in a patient on ruxolitinib. JAAD Case Rep..

[93] Yanamandra U., Sahu K.K., Malhotra P., Varma S. (2014). Photodermatosis secondary to hydroxyurea. BMJ Case Rep..

[94] Leon-Mateos A., Zulaica A., Caeiro J.L., Fabeiro J.M., Calvino S., Peteiro C., Toribio J. (2007). Photo-induced granulomatous eruption by hydroxyurea. J. Eur. Acad. Dermatol. Venereol..

[95] Satanove A., McIntosh J.S. (1967). Phototoxic reactions induced by high doses of chlorpromazine and thioridazine. JAMA.

[96] Rodriguez-Pazos L., Sanchez-Aguilar D., Rodriguez-Granados M.T., Pereiro-Ferreiros M.M., Toribio J. (2010). Erythema multiforme photoinduced by statins. Photodermatol. Photoimmunol. Photomed..

[97] Montanaro S., Lhiaubet-Vallet V., Iesce M.I., Previtera L., Miranda M.A. (2009). A mechanistic study on the phototoxicity of atorvastatin: Singlet oxygen generation by a phenanthrene-like photoproduct. Chem. Res. Toxicol..

[98] Tsai K.C., Yang J.H., Hung S.J. (2017). Fenofibrate-induced photosensitivity—A case series and literature review. Photodermatol. Photoimmunol. Photomed..

[99] Kastalli S., El Aidli S., Chaabane A., Amrani R., Daghfous R., Belkahia C. (2009). Photosensitivity induced by metformin: A report of 3 cases. Tunis. Med..

[100] Dogra S., Kanwar A.J. (2003). Clopidogrel bisulphate-induced photosensitive lichenoid eruption: First report. Br. J. Dermatol..

[101] Bourezane Y., Girardin P., Aubin F., Vigan M., Adessi B., Humbert P., Laurent R. (1996). Allergic contact dermatitis to Zovirax cream. Allergy.

[102] Horio T. (1979). Photosensitivity reaction to dibucaine. Case report and experimental induction. Arch. Dermatol..

[103] Rietschel R.L. (1978). Photocontact dermatitis to hydrocortisone. Contact Dermat..

[104] Horio T. (1975). Chlorpromazine photoallergy. Coexistence of immediate and delayed type. Arch. Dermatol..

[105] Kaidbey K.H., Kligman A.M. (1977). Clinical and histological study of coal tar phototoxicity in humans. Arch. Dermatol..

[106] Bagheri H., Lhiaubet V., Montastruc J.L., Chouini-Lalanne N. (2000). Photosensitivity to ketoprofen: Mechanisms and pharmacoepidemiological data. Drug Saf..

[107] Loh T.Y., Cohen P.R. (2016). Ketoprofen-induced photoallergic dermatitis. Indian J. Med. Res..

[108] Szczurko C., Dompmartin A., Michel M., Moreau A., Leroy D. (1994). Photocontact allergy to oxybenzone: Ten years of experience. Photodermatol. Photoimmunol. Photomed..

[109] Kowalzick L., Ziegler H. (2006). Photoallergic contact dermatitis from topical diclofenac in Solaraze gel. Contact Dermat..

[110] Fernandez-Jorge B., Goday-Bujan J.J., Murga M., Molina F.P., Perez-Varela L., Fonseca E. (2009). Photoallergic contact dermatitis due to diclofenac with cross-reaction to aceclofenac: Two case reports. Contact Dermat..

[111] Canelas M.M., Cardoso J.C., Goncalo M., Figueiredo A. (2010). Photoallergic contact dermatitis from benzydamine presenting mainly as lip dermatitis. Contact Dermat..

[112] Elgezua O.L., Gorrotxategi P.E., Garcia J.G., Nieto J.A.R., Perez J.L.D. (2004). Photoallergic hand eczema due to benzydamine. Eur. J. Dermatol..

[113] Youn J.I., Lee H.G., Yeo U.C., Lee Y.S. (1993). Piroxicam photosensitivity associated with vesicular hand dermatitis. Clin. Exp. Dermatol..

[114] Varela P., Amorim I., Massa A., Sanches M., Silva E. (1998). Piroxicam-beta-cyclodextrin and photosensitivity reactions. Contact Dermat..

[115] Dosik J., Ellman H., Stuart I. (2019). Topical minocycline foam 4%: Results of four phase 1 studies evaluating the potential for phototoxicity, photoallergy, sensitization, and cumulative irritation. J. Immunotoxicol..

[116] Onoue S., Suzuki G., Kato M., Hirota M., Nishida H., Kitagaki M., Kouzuki H., Yamada S. (2013). Non-animal photosafety assessment approaches for cosmetics based on the photochemical and photobiochemical properties. Toxicol. In Vitro.

[117] Scheuer E., Warshaw E. (2006). Sunscreen allergy: A review of epidemiology, clinical characteristics, and responsible allergens. Dermatitis.

[118] Honari G. (2014). Photoallergy. Rev. Environ. Health.

[119] Schauder S., Ippen H. (1997). Contact and photocontact sensitivity to sunscreens. Review of a 15-year experience and of the literature. Contact Dermat..

[120] Santoro F.A., Lim H.W. (2011). Update on photodermatoses. Semin. Cutan. Med. Surg..

[121] Gordon-Thomson C., Gupta R., Tongkao-on W., Ryan A., Halliday G.M., Mason R.S. (2012). 1alpha,25 dihydroxyvitamin D3 enhances cellular defences against UV-induced oxidative and other forms of DNA damage in skin. Photochem. Photobiol. Sci..

[122] Song E.J., Gordon-Thomson C., Cole L., Stern H., Halliday G.M., Damian D.L., Reeve V.E., Mason R.S. (2013). 1alpha,25-Dihydroxyvitamin D3 reduces several types of UV-induced DNA damage and contributes to photoprotection. J. Steroid Biochem. Mol. Biol..

[123] Ma Q. (2013). Role of nrf2 in oxidative stress and toxicity. Ann. Rev. Pharmacol. Toxicol..

[124] Tian F.F., Zhang F.F., Lai X.D., Wang L.J., Yang L., Wang X., Singh G., Zhong J.L. (2011). Nrf2-mediated protection against UVA radiation in human skin keratinocytes. Biosci. Trends.

[125] Kalra S., Knatko E.V., Zhang Y., Honda T., Yamamoto M., Dinkova-Kostova A.T. (2012). Highly potent activation of Nrf2 by topical tricyclic bis(cyano enone): Implications for protection against UV radiation during thiopurine therapy. Cancer Prev. Res. (Phila).

[126] Johansson E.K., Krynitz B., Holmsten M., Klockhoff K., Isaksson Friman E., Xie H. (2018). Severe photo toxicity recalled by docetaxel. Case Rep. Oncol..

[127] Droitcourt C., Le Ho H., Adamski H., Le Gall F., Dupuy A. (2012). Docetaxel-induced photo-recall phenomenon. Photodermatol. Photoimmunol. Photomed..

[128] Camidge D.R. (2001). Methotrexate-induced radiation recall. Am. J. Clin. Oncol..

[129] Jibbe A., Tefft K., Weir G., Aires D., Liu D. (2013). Photo recall reaction following the use of vancomycin. Dermatol. Online J..

[130] Damian D.L. (2010). Photoprotective effects of nicotinamide. Photochem. Photobiol. Sci..

[131] Snaidr V.A., Damian D.L., Halliday G.M. (2019). Nicotinamide for photoprotection and skin cancer chemoprevention: A review of efficacy and safety. Exp. Dermatol..

[132] Damian D.L. (2017). Nicotinamide for skin cancer chemoprevention. Australas. J. Dermatol..

